# Using health technology assessment to set priority, inform target product profiles, and design clinical study for health innovation

**DOI:** 10.1016/j.techfore.2021.121000

**Published:** 2021-11

**Authors:** Yi Wang, Waranya Rattanavipapong, Yot Teerawattananon

**Affiliations:** a Saw Swee Hock School of Public Health, National University of Singapore, Singapore, Singapore; b Health Intervention and Technology Assessment Program, Ministry of Public Health, Nonthaburi, Thailand

**Keywords:** Early health technology assessment, Priority setting, Target product profiles, Uncertainty, Decision support

## Abstract

Early health technology assessment (early HTA) is a useful tool in guiding the innovation development process in medical technology development. However, the application of early HTA is sub-optimal amongst research and development (R&D) communities due to several challenges. In this paper, we presented a case study of application of early HTA by drawing on the experience from a workshop conducted for the Singapore government's medical technology innovation agency. The framework developed can help maximise the chance of the newly developed technology being accepted and widely used. By providing step-by-step guidance, this work aims to translate early HTA into a practical tool and promote the application of early HTA amongst R&D communities.

## Highlights

• Early health technology assessment is useful, but not widely applied.
• Practical steps were provided for conducting early health technology assessment.
• Four concepts of target product profiles were proposed.
• Aim to translate early health technology assessment into a practical tool.

## 1 Introduction

Health technology is an integral part of every health care system's toolkit to tackle challenges and address existing healthcare gaps. These include promoting health, preventing diseases, curing illness, averting morbidity and premature mortality. Proper guidance is required to maximise the chances of successful health technology development and to enhance the efficiency of the research and development (R&D) system, so that health technology innovation can meet the market demand and its cost is not a barrier for access for the target population. By this, the newly developed technology can have a greater impact in terms of improving population health.

The concept of health technology assessment (HTA) was introduced in connection with “evidence-based healthcare” or “evidence-informed decision making”. HTA is a form of policy research to advise decision makers on the potential impact of introducing or withdrawing individual or package of health technologies in terms of health, economic, as well as social and ethical dimensions. A rapid development of new health technologies and limited health budgets have led to the widespread adoption of HTA, first in the US, Europe, eventually spreading to the entire world (Banta, 2003). At present, the main focus of HTA is to promote effective and efficient health care systems. As such, cost-effectiveness analysis is one of the most commonly used methods in informing coverage decisions and price negotiation (Iandolo et al., 2018).

Most HTA agencies are part of the governments supporting health benefit package development for universal healthcare coverage (Glassman et al., 2012; Teerawattananon et al., 2019). It has been argued that HTA is being performed at too late a stage, as investors including medical companies and government innovation agencies have already invested both time and resources in a health technology before being informed by HTA agencies whether the health technology is worthwhile of support by public health plans (Ijzerman and Steuten, 2011). As a result, there is increasing interest from the health technology development community to reversely apply HTA to inform product development (Ijzerman et al., 2017). This is to ensure that the final product meets the demand of patients, health professionals and decision makers, which will eventually reduce investment risk. This concept calls for early HTA as opposed to traditional HTA, which is usually applied after market approval by Food and Drug Administration-like regulatory agencies.

Nevertheless, there are several challenges of early HTA (Ijzerman et al., 2017). These include disconnection between the R&D community and government-owned HTA agencies; lack of awareness of early HTA and its potential amongst the R&D community; lack of standard methodological approach and guidelines for early HTA; and no concrete example of successful implementation of early HTA. This paper draws on our experience of conducting an early HTA workshop for the Singapore government's medical technology innovation agency (Biodesign). The workshop took place on May 27, 2019 and introduced the concept of early HTA to its fellows, which are a group of multidisciplinary innovators, using a simulated case study to illustrate the potential of early HTA in setting R&D priorities, determining target product profiles, and designing a clinical study for a new health innovation.

## 2 Background

### 2.1 Current landscape of early HTA

Most of the initial work on early HTA was in the context of iterative economic evaluation of a medical product at an early stage (Ijzerman et al., 2017). Later, the role of early HTA was extended to inform product profile development, R&D decisions, research decisions, and uncertainty management. Currently, there is no agreed-upon theoretical framework for early HTA and there is little specific guidance on conducting early HTA. One proposed definition of early HTA is “all methods used to inform industry and other stakeholders about the potential value of new medical products in development, including methods to quantify and manage uncertainty” (Ijzerman et al., 2017). Early HTA should be conducted during the conceptual stage, where decisions in R&D investment and the features of the technologies are adjustable. Various overlapping frameworks were developed and applied in early HTA (Cosh et al., 2007; Fenwick et al., 2006; Retèl et al., 2013). The dynamic nature of the technology development process requires flexibility in the assessment framework. No single method was found to produce the right information to cater to the interests of a variety of stakeholders. Ijzerman and Steuten (2011) gave an overall summary of the commonly used methods in early HTA. Headroom analysis is one of the most commonly used methods to examine the value and viability of a new technology (Markiewicz et al., 2016). Experts’ opinions and health economic modelling can be combined to understand the performance of the new technology (Cao et al., 2013; Kip et al., 2018). Value-of-information (VOI) analyses and scenario analyses were usually suggested to manage uncertainties and to understand the needs for further research (Ades et al., 2004; Retèl et al., 2013; Van Harten and Retèl, 2016; Willan and Pinto, 2005). Other issues, such as theories of diffusion, integration of patients’ preferences, and adopting early health economic models with systems engineering approaches, have also been discussed in the literature (Ijzerman et al., 2017; Ijzerman and Steuten, 2011; Pietzsch and Paté-Cornell, 2008).

Manufacturers and innovators are amongst the main target audiances of early HTA. However, the complex nature of early HTA and the lack of a uniformed framework make it difficult for early HTA to become a practical tool used by manufacturers and innovators (Markiewicz et al., 2014). Most of the demonstrations of early HTA evaluated a previously new technology retrospectively. Prospective case studies in partner with innovators at technology's conceptual stage are lacking. This work aims to fill this gap by drawing the experience from a workshop conducted for the Singapore government's medical technology innovation agency.

### 2.2 The case study: great saphenous vein ablation

The great saphenous vein (GSV) is the most common site of venous reflux worldwide. We modified an existing cost-utility study comparing endovenous procedures, including radiofrequency ablation (RFA) and ultrasound-guided foam sclerotherapy (UGFS), with standard surgery in Thailand to fit into the innovation development context (Siribumrungwong et al., 2016). In a hypothetical scenario, the innovator wants to design a new technology I (RFA) for GSV treatment. Compared with the current standard practice S (standard surgery), the technology I is expected to result in less post-operative pain, fewer complications and shorter times for return to normal activities. At the same time, there is a competitor who has just finished designing a technology C (UGFS), which can be used to treat the same problem. The objective of the innovator is to evaluate and maximise the chance of technology I being successful in the market.

By going through the case study, the authors sought for the readers (potential innovators in real life) to learn how to use early HTA to guide the innovation process. We developed four concepts to calculate and design target product profiles (TPP) for the new technology: a minimally acceptable profile, an acceptable profile, an ideal profile, and a stochastic ideal profile. The rationale and incentives behind TPP are that the innovator would like to develop a health technology that is widely used and eventually positively impacts population health. This means that such a technology must be approved by a regulatory agency (for market entry) and accepted for reimbursement by the government in a publicly financed healthcare setting. Hence, the innovator should align their perspective with that of policymakers who make coverage decisions in order to maximize the chance of getting approval for public reimbursement. Furthermore, we incorporated VOI analyses in the context of early HTA to help guide the subsequent clinical studies to generate necessary clinical evidence for traditional HTA as part of the public reimbursement process in a given hypothetical setting.

## 3 Methods

Readers can refer to the original paper to understand the model and source of data in the original study context (Siribumrungwong et al., 2016). In this case study, the original model was used, but the data inputs for the model were modified for the training purpose and in order to develop the economic model specifically for the TPP analyses.

### 3.1 Model and data

The hypothetical scenario was that the innovator brings new technology I for treating GSV to the market, and aims to be successful in obtaining reimbursement approval from the government and policymakers. The target population for the new product is adults with varicose veins (VV) in GSV. Adults included in the study were those who: (i) had isolated unilateral GSV reflux diagnosed by duplex scan; (ii) had no history of deep vein thrombosis or superficial thrombophlebitis; (iii) had no peripheral arterial occlusive disease; (iv) were not pregnant. Patients with reflux in tributaries were not considered. A cost-utility analysis approach was used to assess the value for money of the new intervention. A one-year time horizon was applied. Two interventions were considered in the study: technology I by the innovator and technology C by the competitor. The comparator was the current stand practice S. The outcome variables included costs and quality-adjusted life-years (QALYs), common outcomes in economic evaluations. We assumed that the government or policymakers take the healthcare system's perspective when making reimbursement-approval decisions. Hence, the innovator took the same perspective to examine the cost-effectiveness of their innovation. This also means that technology cost is a market price.

Table 1 shows the values for all the parameters in the model, including transition probabilities, clinical effectiveness, costs, and utility values used to calculate QALYs. In the hypothetical scenario, for the current standard practice S, the values for the parameters were adopted from existing studies. For technology C, the values of the parameters were estimated using information from the competitor's road show, a product launching event, and information gathered from the market research team. For technology I, the values of parameters were the target values set by the innovator after searching the literature, examining existing technologies and meeting with different stakeholders.

**Table 1:** Input Parameter Values.

Parameter name	Description	Value	Distribution	SE	Alpha	Beta
*Transitional probabilities*						
Prob_wound_S	Probability of wound complications after S	0.15	beta	0.07	4	23
Prob_failure_S	Probability of treatment failure after S	0.07	beta	0.05	2	25
Prob_retre_S	Probability of retreatment after S	0.54	beta	0.07	26	22
Prob_retreat_C	Probability of retreatment after C	0.42	beta	0.06	27	37
Prob_retreat_I	Probability of retreatment after I	0.39	beta	0.06	25	39
*Effectiveness*						
RR_wound_C	Relative risk reduction in wound infection (C vs S)	0.30	gamma	0.15	4	0.08
RR_wound_I	Relative risk reduction in wound infection (I vs S)	0.30	gamma	0.15	4	0.08
RR_failure_C	Relative risk of treatment failure (C vs S)	1.30	gamma	0.43	9	0.14
RR_failure_I	Relative risk of treatment failure (I vs S)	1.25	gamma	0.42	9	0.14
*Cost*						
Cost_treatment_S	Treatment cost of S	1300	gamma	130	100	13
Cost_treatmentFail_S	Treatment cost of S in patients who failed	1900	gamma	190	100	19
Cost_wound_S	Treatment cost of S in patients with wound infection	1600	gamma	160	100	16
Cost_woundFail_S	Treatment cost of S in patients with wound infection and failed	2000	gamma	200	100	20
Cost_treatment_C	Treatment cost of C	2700	gamma	270	100	27
Cost_treatmentFail_C	Treatment cost of C in patients who failed	3200	gamma	320	100	32
Cost_wound_C	Treatment cost of C in patients with wound infection	3100	gamma	310	100	31
Cost_woundFail_C	Treatment cost of C in patients with wound infection and failed	3500	gamma	350	100	35
Cost_retreat	Retreatment cost	1000	gamma	100	100	10
Cost_retreat_I	Retreatment cost of I	1100	gamma	110	100	11
Cost_treatment_I	Treatment cost of I	3500	gamma	350	100	35
Cost_treatmentFail_I	Treatment cost of I in patients who failed	3800	gamma	380	100	38
Cost_wound_I	Treatment cost of I in patients with wound infection	3700	gamma	370	100	37
Cost_woundFail_I	Treatment cost of I in patients with wound infection and failed	4000	gamma	400	100	40
*Utility*						
Utility_success_S	Utility of patients who are successful after S	0.68	beta	0.01	1479	696
Utility_fail_S	Utility of patients who failed after S	0.50	beta	0.01	1250	1250
Utility_retreatFail_S	Utility of patients who failed after S and undergoing retreatment	0.44	beta	0.01	1084	1379
Utility_wound_S	Utility of patients who get wound infections after S success	0.48	beta	0.01	1198	1297
Utility_woundFail_S	Utility of patients who get wound infections after S fail	0.41	beta	0.01	991	1427
Utility_woundRetreat_S	Utility of patients who get wound infections after S fail and undergo retreatment	0.44	beta	0.01	1084	1379
Utility_success_C	Utility of patients who are successful after C	0.88	beta	0.01	928	127
Utility_fail_C	Utility of patients who failed after C	0.50	beta	0.01	1250	1250
Utility_retreatFail_C	Utility of patients who failed after C and undergoing retreatment	0.45	beta	0.01	1113	1361
Utility_wound_C	Utility of patients who get wound infections after C success	0.72	beta	0.01	1451	564
Utility_woundFail_C	Utility of patients who get wound infections after C fail	0.48	beta	0.01	1198	1297
Utility_woundRetreat_C	Utility of patients who get wound infections after C fail and undergo retreatment	0.47	beta	0.01	1170	1320
Utility_success_I	Utility of patients who are successful after I	0.93	beta	0.01	605	45
Utility_fail_I	Utility of patients who failed after I	0.55	beta	0.01	1361	1113
Utility_retreatFail_I	Utility of patients who failed after I and undergoing retreatment	0.47	beta	0.01	1170	1320
Utility_wound_I	Utility of patients who get wound infections after I success	0.76	beta	0.01	1385	438
Utility_woundFail_I	Utility of patients who get wound infections after I fail	0.49	beta	0.01	1224	1274
Utility_woundRetreat_I	Utility of patients who get wound infections after I fail and undergo retreatment	0.48	beta	0.01	1224	1274

Notes: S represents the current standard practice. C represents the competitor's technology. I represents the innovator's technology.

### 3.2 Overview of analysis

#### 3.2.1 Cost-utility analysis

**Fig. 1:**
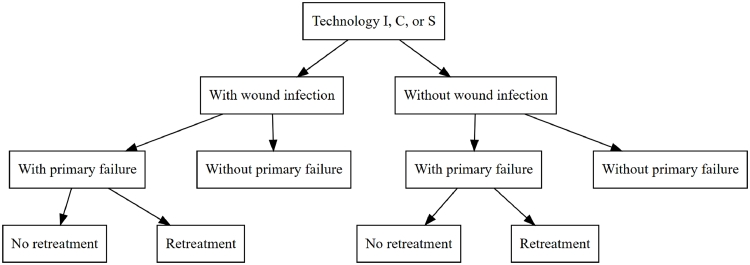
Decision Tree. Notes: S represents the current standard practice. C represents the competitor's technology. I represents the innovator's technology.

A cost-utility analysis was conducted to evaluate the cost-effectiveness comparing technology I, technology C and the current standard practice S. A decision tree model was used (Fig. 1). The incremental cost-effectiveness ratio (ICER) was calculated, as the incremental cost per incremental QALY, and reported to reflect the value for money of the interventions.

Uncertainty analyses were performed to take account for parameter uncertainty. A probabilistic sensitivity analysis (PSA) was performed, which involved the sampling of all model parameters from their statistical distributions. The results of the PSA were presented in the form of cost-effectiveness acceptability curve (CEAC) and cost-effectiveness acceptability frontier (CEAF). A one-way deterministic sensitivity analysis (DSA) was performed, by varying a parameter between a lower bound and an upper bound, while all the other parameters were held constant. The results of the DSA were presented using the tornado diagrams.

#### 3.2.2 Target product profile

**Fig. 2:**
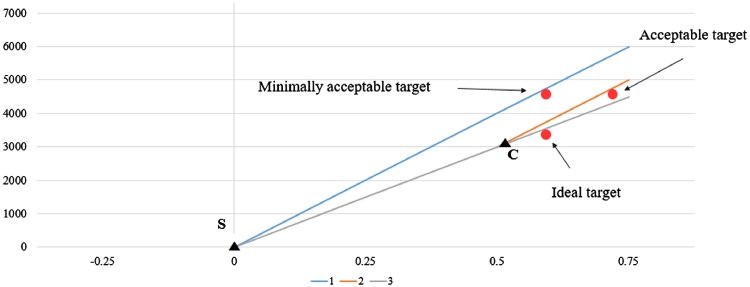
Targeted Product Profile Criteria. Notes: This figure demonstrates the minimally acceptable target, acceptable target, and ideal target using a cost-effective plane. The x-axis is incremental outcome measure (e.g.: QALYs) and the y-axis is incremental cost. S represents the standard practice. Line 1 represents the cost-effective threshold used in the society. Line 2 passes through C, the competitor's technology, and is parallel to the line 1. Assume the competitor's technology is cost-effective and represents the current best practice. The red points represent the innovator's technology. To satisfy the minimally acceptable target, the innovator's technology should be below the cost-effective threshold line 1. To satisfy the acceptable target, the innovator's technology should be below line 2. To satisfy the ideal target, the innovator's technology should be below line 3. (For interpretation of the references to colour in this figure legend, the reader is referred to the web version of this article.)

TPPs were developed in order to determine and maximise the probability of the product being successful in the market. We considered four criteria: a minimally acceptable target, an acceptable target, an ideal target, and a stochastic ideal target. Fig. 2 demonstrates the first three criteria using a cost-effectiveness plane, with the x-axis representing incremental outcome measure (i.e.: QALYs) and y-axis displaying incremental costs. Line 1 represents the cost-effectiveness threshold used in the society. The minimally acceptable target was defined as being cost-effective compared with the current standard practice. To satisfy this criterion, innovation I should be below the cost-effectiveness threshold line 1. The acceptable target was defined as cost-effective compared to the current best practice. For demonstration purpose, let's assume technology C is the best currently available technology. Line 2 passes through C and is parallel to line 1. To satisfy the acceptable target, innovation I should be below line 2. The ideal target was defined as the best amongst all the products that dominate the market. The ICER comparing innovation I with current standard practice S should be smaller than the ICER comparing alternative technology C with current standard practice S. In Fig. 2, to satisfy this criterion, Innovation I should be below line 3.

We proposed two ways to calculate the minimally acceptable target, acceptable target and ideal target. The first way is to use the mean value of parameters to calculate the desired product profile, referred as deterministic targets. The second way is to use the distribution of the parameters and take the average of the results from the probabilistic model to calculate the desired product profile, referred as probabilistic-average targets. The deterministic method and the probabilistic-average method give the same results in a linear model.

The stochastic ideal target was defined as the new technology being cost-effective with at least a predetermined probability. In this study, we considered 70%, 80%, and 90%. Taking 90% as an example, the innovator's new technology I would achieve the stochastic ideal target if it is cost-effective 90% of the time compared to technology C and S.

In our analysis, first, we modified one parameter at a time to achieve the proposed targets, which is often applied in previous studies as a headroom analysis (Cao et al., 2013; Markiewicz et al., 2016). The stochastic ideal target is our new concept for TPPs, although the underlying concept, PSA, is not new. To the best of our knowledge, no study has applied these criteria before when calculating TPPs. Second, we selected two parameters and did two-way analysis considering different targets proposed in our study. This allows the innovator to achieve TPPs with more flexibility.

#### 3.2.3 Value-of-information analysis

A VOI analysis is an analytical approach to understand the value of conducting additional research in order to reduce uncertainties in decision-making (Claxton and Sculpher, 2006). The literature on VOI analyses for supporting coverage decision of a finished product is abundant with practical guidance on VOI calculation being available (Fenwick et al., 2020; Rothery et al., 2020). However, applications of VOI in early HTA to inform new technology development are rare. At the early stage of new technology development, the uncertainties in individual parameters are high. VOI analysis is meant to address these uncertainties, which could have broader roles in early HTA.

We applied VOI analyses considering the current product profile, as well as the product profiles satisfying the four of the proposed criteria: deterministic minimally acceptable target, deterministic acceptable target, deterministic ideal target, and stochastic ideal target achieving 90%. First, the expected value of perfect information (EVPI) was calculated using different cost-effectiveness thresholds to determine the opportunity cost of making the wrong decision in adopting new technology or, in other words, the overall value of conducting additional research to reduce uncertainties. Second, the expected value of partial perfect information (EVPPI) was calculated to estimate the value of reducing uncertainties for each individual parameter. The results can be used to prioritise the parameters for which further research would be worthwhile. Third, for selected parameters, the expected value of sample information (EVSI) was calculated to inform the optimal sample size if the corresponding clinical research is to be conducted.

A hypothetical cost-effectiveness threshold (or ceiling threshold) of 7000 USD per QALY was considered in this setting to interpret the results and explore further analyses i.e.: TPP, EVPI and EVPPI. Detailed methodology, including R® commands are provided in the Supplementary Material.

## 4 Results

### 4.1 Cost-utility analysis

**Fig. 3:**
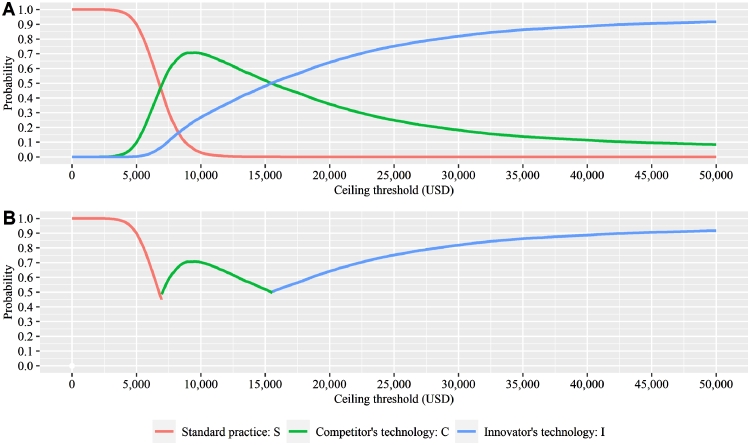
Cost-effectiveness Acceptability Curve and Cost-effectiveness Acceptability Frontier. Notes: Panel A shows the cost-effectiveness acceptability curve. Panel B shows the cost-effectiveness acceptability frontier. .

**Fig. 4:**
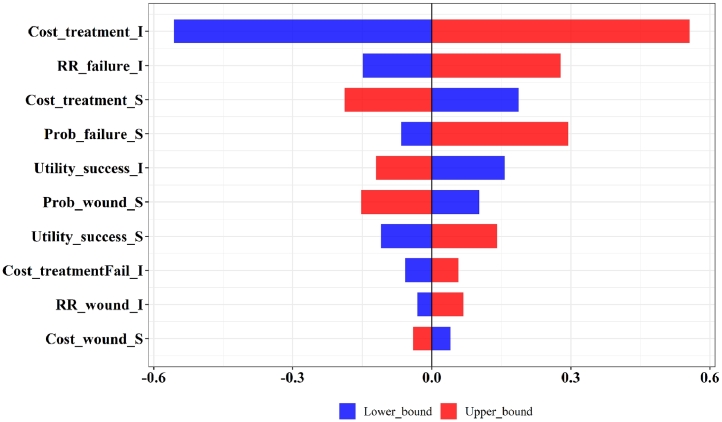
One-way sensitivity analysis of I versus S. Notes: Full descriptions of the parameters can be obtained from Table 1. The top 10 parameters are shown in this figure. S represents the current standard practice. I represents the innovator's technology.

**Fig. 5:**
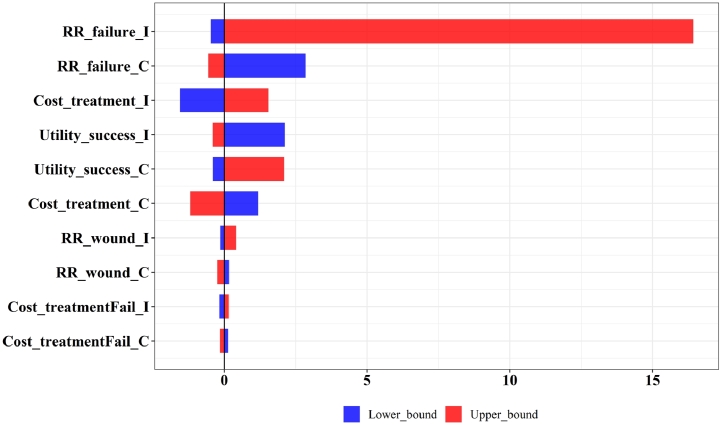
One-way sensitivity analysis of I versus C. Notes: Full descriptions of the parameters can be obtained from Table 1. The top 10 parameters are shown in this figure. C represents the competitor's technology. I represents the innovator's technology.

Table 2 shows the results of the deterministic cost-utility analysis. Comparing with the current standard practice S, the competitor's technology C was cost-effective, with an ICER of 6888 USD/QALY. The innovator's technology I was not cost-effective, with an ICER of 8596 USD/QALY. If the competitor's technology was adopted and became the standard practice by the time that the innovator launched technology I, comparing I to C, the ICER was 15,442 USD/QALY. The results suggest that the innovator's current technology I is unlikely to become the standard practice or obtain reimbursement from the government. The CEAC and CEAF are shown in Fig. 3. The probability of the innovator's technology I being cost-effective increased with the ceiling threshold. The results from DSA comparing the innovator's technology I to the current standard practice S and the competitor's technology C are shown in Fig. 4, Fig. 5. The top 10 most influential parameters, in terms of effect size, are presented.

**Table 2:** Cost-Utility Analysis Results.

Results (Deterministic analysis)				
	Standard Practice S	The Competitor's Technology C	The Innovator's Technology I	I vs C
Cost (USD)	1423	2801	3573	
QALYs (year)	0.64	0.84	0.89	
Incremental cost		1379	2150	771
Incremental QALYs		0.20	0.25	0.05
ICER (USD/QALY gained)		6888	8596	15,442

### 4.2 Target product profile

Five parameters of the innovator's technology I were selected to design the TPP. These five parameters were amongst the top ten parameters in the DSA when comparing the innovator's technology I with the competitor's technology C and the current standard practice S. The results are presented in Table 3. The baseline values correspond to the current product profile. To make the innovator's technology I represent good value for money, its treatment cost needs to be 3041 USD using the deterministic minimally acceptable target, 3016 USD using the deterministic acceptable target, and 3009 USD to achieve the deterministic ideal target. The probabilistic-average targets required slightly lower values compared to the corresponding deterministic targets. To achieve the stochastic ideal target with 70%, 80%, and 90%, the cost of technology I needs to be 2630 USD, 2470 USD, and 2230 USD, respectively.

**Table 3:** Targeted Product Profile.

Profile of technology I	Base values	Deterministic minimally acceptable target	Deterministic acceptable target	Deterministic ideal target
Treatment cost of I (USD)	3500	3041	3016	3009
Treatment cost of I in patients who failed (USD)	3800	−979	−1248	−1315
RR of treatment failure comparing I with S	1.25	−0.36	−0.44	−0.49
RR of wound infection comparing I with S	0.3	−1.74	−1.86	−1.92
Utility of patients who are successful under I	0.93	0.996	1	1.002

Profile of technology I	Base values	Probabilistic-average minimally acceptable target	Probabilistic-average acceptable target	Probabilistic-average ideal target

Treatment cost of I (USD)	3500	3017	3006	3003
Treatment cost of I in patients who failed (USD)	3800	−958	−1062	−1088
RR of treatment failure comparing I with S	1.25	−0.35	−0.39	−0.40
RR of wound infection comparing I with S	0.3	−1.89	−1.93	−1.96
Utility of patients who are successful under I	0.93	0.999	1.001	1.002

Profile of technology I	Base values	Stochastic ideal target (70%)	Stochastic ideal target (80%)	Stochastic ideal target (90%)

Treatment cost of I (USD)	3500	2630	2470	2230
Treatment cost of I in patients who failed (USD)	3800	−7100	−11,700	−22,100
RR of treatment failure comparing I with S	1.25	−2.50	−4	−7.40
RR of wound infection comparing I with S	0.3	−5.10	−7.40	−12.30
Utility of patients who are successful under I	0.93	1.054	1.077	1.112

Notes: S represents the current standard practice. C represents the competitor's technology. I represents the innovator's technology. RR stands for relative risk.

For the treatment cost of technology I in patients who failed, the relative risk (RR) of treatment failure and RR of wound infection, negative values, which are beyond the lower limit, are required to meet the targets. For the utility of patients who are successful after undertaking technology I, values larger than 1, which are beyond the upper limit of health utility values, are required to meet certain targets.

**Fig. 6:**
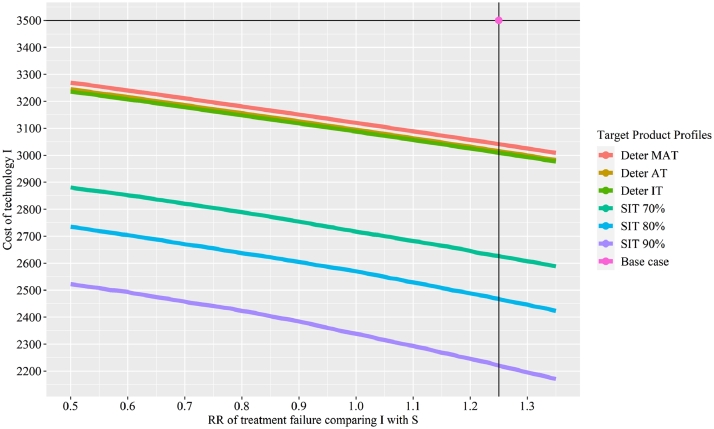
Target Product Profiles – Two-way Analysis. Notes: RR stands for relative risk. S represents the current standard practice. I represents the innovator's technology. Deter MAT stands for deterministic minimally acceptable target. Deter AT stands for deterministic acceptable target. Deter IT stands for deterministic ideal target. SIT stands for stochastic ideal target. .

In Fig. 6, we showed results of two-way analysis considering cost of technology I and RR of treatment failure comparing technology I with S, top two parameters of technology I based on one-way sensitivity analysis. The baseline values were presented by the dot labelled as “Base case”. Three deterministic targets and stochastic ideal targets with 70%, 80% and 90% were considered. With a lower RR of treatment failure comparing technology I with S, a higher cost of technology I can be accepted.

### 4.3 Value-of-information analysis

We considered five scenarios when conducting VOI analysis with the mean cost of innovator's technology being: 3500 USD under the baseline case, 3041 USD under the deterministic minimally acceptable target, 3016 USD under the deterministic acceptable target, 3009 USD under the deterministic ideal target, and 2230 USD under the stochastic ideal target at 90%. Under each scenario, the standard errors (SEs) remain the same as the TPPs focus on the product profile, only affecting the mean values of the parameters.

**Fig. 7:**
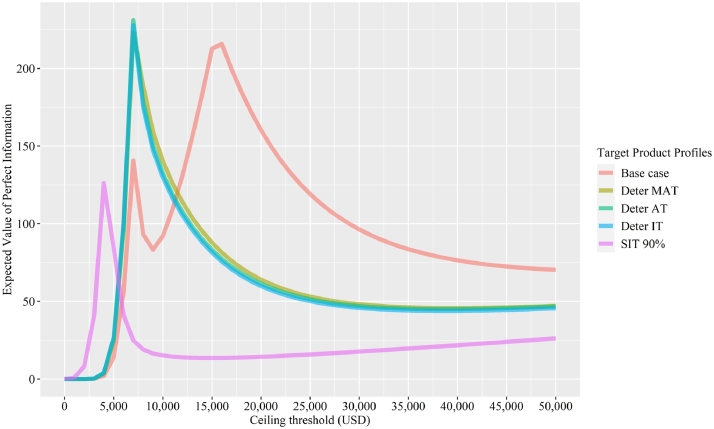
EVPI versus Ceiling Threshold per QALY Gained. Notes: The unit of EVPI is USD per patient. Deter MAT stands for deterministic minimally acceptable target. Deter AT stands for deterministic acceptable target. Deter IT stands for deterministic ideal target. SIT stands for stochastic ideal target. QALY stands for quality-adjusted life-year.

Fig. 7 shows the curves of EVPI versus the ceiling threshold. Five curves were plotted corresponding to the five scenarios. Under the baseline case, there are two peaks for the EVPI curve. The corresponding thresholds are around the thresholds where the CEAC curves intersected in Fig. 3. For the deterministic minimally acceptable target, deterministic acceptable target, and deterministic ideal target scenarios, the three curves are close to each other in our example as the costs of technology I are close to each other. In the stochastic ideal target scenario, the values for the EVPI are much smaller at the ceiling threshold of 7000 USD and peak at a lower ceiling threshold, around 4000 USD. This result is not surprising as the stochastic ideal target takes decision uncertainties into consideration.

Table 4 shows the EVPPI for the five scenarios using the ceiling threshold value of 7000 USD/QALY. The parameters with top five highest EVPPI under each scenario are highlighted in bold. Under the baseline scenario, the top five parameters are: the cost of technology C (83.51 USD per patient), the probability of failure of treatment S (40.2 USD per patient), the RR of failure comparing technology C with S (34.34 USD per patient), the cost of treatment S (30.68 USD per patient), and the probability of wound infection of treatment S (21.49 USD per patient). The EVPPI of the cost of technology I is 9.52 USD per patient. The EVPPI of the RR of failure for technology I compared with S is 0 USD per patient. Under the three scenarios where the deterministic minimally acceptable target, deterministic acceptable target, and deterministic ideal target were satisfied, the cost of technology I and the RR of failure for comparing technology I with S were amongst the top five parameters with the highest EVPPI. In the stochastic ideal target scenario, the EVPPI for majority of the parameters become 0, with only four parameters having small positive values.

**Table 4:** EVPPI Results.

Parameter name	Base case	Deterministic minimally acceptable target	Deterministic acceptable target	Deterministic ideal target	Stochastic ideal target (90%)
Prob_wound_S	**21.49**	21.56	21.42	17.59	0
Prob_failure_S	**40.2**	**39.87**	**44.31**	**43.59**	**0.35**
Prob_retre_S	0	0	0	0	0
Prob_retreat_C	0.003	0.003	2.86	0.99	0
Prob_retreat_I	0	0.006	3.43	1.50	0
RR_wound_C	5.33	5.36	12.39	9.63	0
RR_wound_I	0	5.12	15.31	13.3	0
RR_failure_C	**34.34**	**34.35**	**43.46**	**41**	0
RR_failure_I	0	**34.85**	**46.35**	**44.43**	**0.002**
Cost_treatment_S	**30.68**	30.32	30.65	28.28	0
Cost_treatmentFail_S	0.06	0.06	0.06	0.01	0
Cost_wound_S	1.67	1.64	1.63	0.97	0
Cost_woundFail_S	0	0	0	0	0
Cost_treatment_C	**83.51**	**82.62**	**93.31**	**90.54**	**0.04**
Cost_treatmentFail_C	3.47	3.46	11.08	8.55	0
Cost_wound_C	0.26	0.27	5.01	2.82	0
Cost_woundFail_C	0	0	0.57	0	0
Cost_retreat	0	0	1.50	0.12	0
Cost_retreat_I	0	0	1.49	0.17	0
Cost_treatment_I	9.52	**110.70**	**121.38**	**118.53**	**2.14**
Cost_treatmentFail_I	0	4.23	12.59	10.33	0
Cost_wound_I	0	0.35	6.01	3.86	0
Cost_woundFail_I	0	0	0.62	0.001	0
Utility_success_S	0.02	0.02	0.02	0.02	0
Utility_fail_S	0	0	0	0	0
Utility_retreatFail_S	0	0	0	0	0
Utility_wound_S	0.03	0.03	0.03	0.006	0
Utility_woundFail_S	0	0	0	0	0
Utility_woundRetreat_S	0	0	0	0	0
Utility_success_C	14.9	14.68	24.09	21.55	0
Utility_fail_C	0	0	1.40	0.11	0
Utility_retreatFail_C	0	0	1.01	0.02	0
Utility_wound_C	0	0	1.13	0.04	0
Utility_woundFail_C	0	0	0.06	0	0
Utility_woundRetreat_C	0	0	0.04	0	0
Utility_success_I	0	14.35	24.21	21.91	0
Utility_fail_I	0	0	1.42	0.11	0
Utility_retreatFail_I	0	0	0.90	0.008	0
Utility_wound_I	0	0	1.13	0.04	0
Utility_woundFail_I	0	0	0.06	0	0
Utility_woundRetreat_I	0	0	0.04	0	0

Notes: Full descriptions of the parameters can be obtained from Table 1. The unit of EVPPI is USD per patient. S represents the current standard practice. C represents the competitor's technology. I represents the innovator's technology.

We selected two parameters with high EVPPIs, the cost of innovator's technology I and the RR of failure comparing technology I to S, when conducting EVSI calculation. The results were presented in Table 5 and Table 6. As the sample size from the additional research increases, the EVSI of cost of technology I increases at a decreasing rate. For a fixed sample size, the EVSIs are higher for the scenarios which satisfy the minimally acceptable target, acceptable target, and ideal target. The EVSI is close to 0 under the stochastic ideal target. This pattern is consistent with the results of EVPPI. The RR of failure comparing technology I to S is of clinical interest. The innovator is likely to conduct studies examining this throughout the innovation process. Table 6 shows the results of the EVSI analysis. We considered the same five TPPs. The pattern of the results matches with the results of the EVPPI analyses.

**Table 5:** EVSI - Cost of Technology I.

Sample size	Base case	Deterministic minimally acceptable target	Deterministic acceptable target	Deterministic ideal target	Stochastic ideal target (90%)
10	0.05	48.93	59.78	57.78	0
20	0.49	64.92	75.96	73.75	0.05
30	1.23	74.51	85.04	82.53	0.12
40	1.9	80.01	90.99	88.48	0.26
50	2.42	84.01	95.25	92.74	0.39
60	3.02	87.39	98.55	96.04	0.55
80	3.85	91.55	102.85	100.8	0.86
100	4.51	94.31	105.63	103.85	1.02
120	4.97	96.49	107.8	105.6	1.16
140	5.27	97.95	109.31	107.28	1.3
160	5.57	99.58	110.92	108.54	1.31
180	5.86	100.39	111.75	109.59	1.54
200	6.08	101.35	112.75	110.36	1.58

Notes: The unit of EVSI is USD per patient. I represents the innovator's technology.

**Table 6:** EVSI - Relative Risk of Failure of I Comparing to S.

**Total sample size(Assume 50% in treatment group and 50% in control group)**	Base case	Deterministic minimally acceptable target	Deterministic acceptable target	Deterministic ideal target	Stochastic ideal target (90%)
10	0	0.42	4.19	2.91	0
20	0	0.94	6.28	4.54	0
30	0	1.57	7.99	6	0
40	0	2.28	9.43	7.31	0
50	0	2.98	10.75	8.56	0
60	0	3.72	12.01	9.76	0
80	0	5.07	13.9	11.77	0
100	0	6.23	15.34	13.81	0
120	0	7.3	16.6	15.52	0
140	0	8.37	17.93	16.88	0
160	0	9.18	18.83	18.03	0
180	0	9.99	19.78	19.59	0
200	0	10.73	20.6	20.54	0

Notes: The unit of EVSI is USD per patient. S represents the current standard practice. I represents the innovator's technology.

## 5 Discussion

At present, more and more governments in high- and middle-income countries as well as large, medium and small enterprises are investing in medical technology R&D. Although early HTA has great potential to make a link between R&D, market approval and public reimbursement of health innovation, there is no concrete example of how early HTA can be applied to inform product development in real-life. This study makes a closer stride towards this by illustrating step-by-step guidance for how economic evaluation including VOI analysis can be helpful for this purpose. This includes the determination of TPPs, prioritisation of clinical and economic measures in clinical studies and value-based sample size calculations. This study can help horizon scanning institutes, HTA practitioners, R&D funders and technology developers to minimise risk and, thus, enhance efficiency in R&D as well as improving accessibility of new technology by shortening the timeline from development to delivery and reducing the cost of new technology.

In our example, the innovator's technology I with the original product profile was not cost-effective compared to the current standard practice, S. It was also not as competitive as the competitor's technology C. Hence, the innovator's technology I is unlikely to be reimbursed by the government. Resources should not be invested in technology I with the original product profile. Deterministic targets, probabilistic-average targets, and stochastic ideal target were proposed to guide innovators in designing TPPs and making their technology more competitive. The appropriate targets should be chosen considering policymakers’ decision-making process. The profiles of a technology that are likely to be approved and reimbursed can be identified to inform product development. Deterministic targets and probabilistic-average targets are based on similar concepts with different calculation methods. Both do not consider decision uncertainty. In a non-linear model, deterministic targets and probabilistic-average targets are likely to be different. Deterministic targets are easier to calculate which only require the mean values of parameters whereas both probabilistic-average targets and stochastic ideal target require the parameter distributions for simulations. As such, at the early stage of innovation process with limited information available, deterministic targets can be established as a starting point.

Considering the dynamic environment of medical innovation industry, we proposed three different terms for deterministic targets and probabilistic-average targets. Without a competitor, innovators can consider only minimally acceptable target by comparing their innovation with the current standard practice. However, acceptable target and ideal target become relevant when the market is crowded with potential competitors with alternative cost-effective technologies as compared to the current stand practice. For example, if competitors’ technology is expected to be cost-effective compared to the current standard practice, acceptable target ensures innovators’ technology being cost-effective compared to competitors’ technology. If the policymakers compare innovators’ technology and competitors’ technology to the current standard practice at the same time and choose one of the technologies to adopt, ideal target ensures innovators’ technology being the best policy choice. As a result, innovators need to do horizon scanning in advance to understand market environment (Hines et al., 2019).

The stochastic ideal target defines the product profile by making the innovation being cost-effective under a predefined probability. We considered the innovator's technology, the competitor's technology and the standard practice together when calculating stochastic ideal target in our case study. However, in practice, innovators may only focus on either the standard practice or competitors’ technology when calculating stochastic ideal target, depending on policy questions. The stochastic ideal target factors uncertainties that policymakers may be risk-averse and only adopt the innovation if the probability of the innovation being cost-effective is high enough. Innovators should consult relevant policymakers and stakeholders to understand the probability to target.

It is not always the case that stochastic ideal target requires a higher standard of TPPs as compared to deterministic targets and probabilistic-average targets. Stochastic ideal target does not guarantee positive expected net benefit. It is possible that an innovation has a large probability of small gains and a small probability of huge losses, resulting in overall negative expected net benefit. In such a scenario, it is possible that the deterministic target and probabilistic-average target reveal more stringent requirements, e.g. lower cost and better performance as compared to the stochastic ideal target. Stochastic ideal target also does not guarantee low VOI. Besides the probability of making wrong decisions, VOI also considers the consequence and the cost of making wrong decisions, which are not captured by stochastic ideal target. Hence, innovators need to understand the decision-making context to select the appropriate targets. Innovators should also not set target that is too ambitious, as that may discourage R&D itself.

The new technology could have many features to be improved which require systematic guidance to set priorities. Based on the results from the DSA, five parameters of technology I were selected for TPP analysis, namely i) treatment cost of technology I, ii) treatment cost of technology I in patients who failed, iii) RR of treatment failure comparing technology I with treatment S, iv) RR of wound infection comparing technology I with treatment S, and v) utility of patients who are successful using technology I. Our findings suggest that unrealistic values were required for four of the parameters to meet the targets, namely treatment cost of technology I in patients’ who failed, RR of treatment failure comparing technology I with treatment S, RR of wound infection comparing technology I with treatment S, and utility of patients who are successful using technology I. In reality, certain features of a technology could be too costly and time-consuming to improve. Innovators can also consider improving multiple features of a technology at the same time in order to achieve the targets. However, the number of combinations of parameters could be overwhelming when considering multiple features together. Innovators can prioritize a group of parameters based on the results from DSA. Then, innovators can consider the group of parameters that have higher impact on the outcome variables, the group of parameters that are easier to improve, or the group of the parameters that have synergies impact. Innovators can also consider using two-way or multi-way sensitivity analyses to set priorities in selecting technology features to be enhanced. For example, our study explored the top two parameters of the innovator's technology I when conducting two-way analysis to determine TPPs.

This work illustrated the difference and importance of using VOI analysis with a probabilistic model compared to the headroom analysis of deterministic model in informing TPPs. Headroom is about the difference from cost-effectiveness; VOI is around the uncertainty in cost-effectiveness. The VOI analysis in this study is used to illustrate the superiority of ‘stochastic ideal target’ compared to other targets set by a popular deterministic approach used by other literatures. Ironically, this study is the first attempt at demonstrating that TPPs derived from headroom analysis using deterministic models are sub-optimal leading to even higher uncertainty in making coverage decisions for new technologies. Our findings suggest that TPPs derived from the PSA and VOI analysis using probabilistic model is an optimal choice because it reduces uncertainty and ensures robustness in the decision-making process for technology adoption. Our results suggest that under the stochastic ideal target at 90% probability of being cost-effectiveness, the values for EVPI, EVPPI, and EVSI are all very low. In such a case, further research may not be required and this helps accelerate the reimbursement approval process. Nevertheless, VOI analysis remains crucial in early HTA using stochastic approach when i) the predetermined probability is set at much less than 90% used in this case study and ii) it is impossible for innovators to achieve the stochastic ideal target. In such cases, recognizing the parameters’ uncertainties and understanding the impact at the early stage of technology development can help plan further research and accelerate the decision-making process for technology adoption. Innovators can balance between the TPPs to target and the uncertainties to address using additional research to optimize the technology development process. If innovators consider a less ambitious TPP as being preferable, innovators can select parameters with high EVPPIs for EVSI calculation, and plan the required research at an early stage.

It is important to note that the TPPs defined at an early stage of the R&D process can be changed once the technology is further developed. Early HTA should not be seen as a one-off process. It should be applied iteratively along the innovation process. For example, the uncertainty is usually high at the early stage, and the quality of the model and parameters used can be relatively low. The quality of evidence, e.g.: TPPs, will also be less robust. If the innovation is at the late stage where the technology is about to be tested in a clinical study, early HTA is likely to use an accurate model and high-quality parameters. In this case, the EVSI analysis can be used to optimise the required clinical study. For example, to examine the clinical performance of the innovator's technology I, a study examining the RR of failure of I compared to current stand practice S is to be conducted. Suppose the minimum samples size of 40 is required considering the significance level, power, dropout rate, and potentially other issues. EVSI analysis can inform whether the cost of collecting effectiveness data using 40 samples is economically justifiable. As suggested in Table 5 and Table 6, the EVSI is low under the stochastic ideal target. Sample sizes larger than 40 are unlikely to be justifiable. However, if the current technology is under the acceptable target, the EVSI is relatively high; then the innovator can consider recruiting more than 40 samples i.e.: 40+*N*. The expected net benefit of sampling (ENBS) analysis can be used to optimise the number of additional sample(s) or N, by not collecting more data than would be required by the HTA agencies in the given setting(s) (Ades et al., 2004). As a result, innovators can maximise the value of investment in clinical research of a new technology, resulting in a lower cost of developing new technology as a whole. This would lead to more affordable technology to be developed for populations of need in resource-limited settings.

Worldwide, billions of dollars are invested in health technology development (Dorsey et al., 2010). Applying early HTA to guide the process can save cost, accelerate market access, and promote more equitable services. From the societal perspective, health benefit from medical innovation can be maximized. From innovators’ perspective, a more efficient innovation process can be achieved and the likelihood of having successful technologies can be maximized, which can increase the confidence of investors and encourage further investment in medical R&D. In this study, we tailored a real-world study and converted it into a case study to show step-by-step guidance in applying early HTA. We proposed several targets for product profiles which can guide innovators to design TPPs. We highlighted the link and trade-off between TPPs and uncertainties. An ambitious TPP can reduce the VOI and the need of doing additional research. In a scenario where the ambitious TPP cannot be achieved or is too costly to achieve, innovators should consider the need for additional research embedded in the innovation process accordingly.

There are several limitations to this study. First, similar to traditional HTA, stakeholder involvement in the process of early HTA is crucial. The stakeholders can help identify competing technologies to be included in the analysis, inform decision-making criteria used in market approval, coverage decisions and appropriate product profile targets to use, as well as verify parameters used in the analysis. Because this study is a hypothetical case, there was no stakeholder consultation in the process of early HTA. However, this study was conducted as part of a collaboration between Saw Swee Hock School of Public Health, Health Intervention and Technology Assessment Program (HITAP) and Biodesign. The methodological approach presented in this paper alongside hand-on exercises on early HTA were introduced to the Biodesign fellows of the 2019/2020 cohort. The post-workshop survey reflects that most participants perceived early HTA for technology development as useful, appreciated the approach and enjoyed the hands-on learning. A series of follow-up consultations were provided to the fellows for applying early HTA in real-life technology development.

Second, the study assumed that the innovator aligns the perspective with policymakers. The costs are from policymakers’ perspective representing the maximum price they are willing to pay for. For the innovator, the costs of technology I are related to the price they can charge. The innovator can compare the price with the cost of R&D, production, distribution, and application of their innovation to understand the profitability. For the innovator, profitability is another key factor to consider when deciding TPPs. Furthermore, there are risks associated with R&D itself. The innovator may fail to invent the technology with the TPPs. These were not considered in this study.

Third, population EVPI, population EVPPI and ENBS should be used to inform the research decisions and to optimise the sample size for clinical studies (Fenwick et al., 2020). These were not explored in this study. Innovators need to estimate the size of patient pool and the time horizon of the impact for their technology to understand the cost and health impact of their innovation at the population level.

Fourth, a certain study is required to demonstrate the safety of the new technology. The design of this safety study is out of the scope of the VOI analysis in general. Moreover, depending on the stage of the innovation process and the quality of data and information, innovators should use the results of VOI analyses carefully. At an early stage of innovation, when the quality of information is low, innovators can use the results from VOI to prioritise further research. Innovators should be more conservative about deciding the sample size of the study, i.e.: using a larger sample size. At the later stage of innovation process, where the quality of information is high, innovators should use the results of VOI to justify and optimise the sample size of subsequent clinical research. However, how to assess the quality of evidence and information is out of the scope of this study.

Fifth, in this study, the TPP calculation considered improving the average performance of the technology focusing on the mean value of the parameters. However, at the early stage of innovation, the performance, e.g.: clinical effectiveness, of the technology could also vary. Stability of the performance of the technology could be a product profile to consider. Furthermore, the administrative process in applying the technology and the treatment regime could also be uncertain, which can affect the amount that the policymakers have to reimburse. To address this issue, a certain feature of the technology can be modelled using a distribution, e.g.: with a mean parameter and a variance parameter. However, it is important to distinguish this variance parameter, which should be addressed considering TPP, with the SEs, which should be addressed in the VOI analysis. Further research can be conducted to address this issue. More elaboration can be found in the Supplementary Material.

Sixth, we tweaked some distributions for calculating EVSI. The cost of innovator's technology I followed Gamma distribution in the original study. However, EVSI calculation of cost with Gamma distribution is complicated and time-consuming. We used Log-normal distribution to approximate the Gamma distribution by matching the mean and variance. More information can be found in the Supplementary Material. From a technical perspective, researchers and innovators should consider both appropriateness of the distributions in modelling the parameters, as well as the ease of calculation and simulation.

## 6 Conclusion

With growing demand for medical technology innovation, early HTA has great potential to guide the innovation process and maximises the benefits of innovation. However, due to the complex nature of early HTA and disconnection between academia and R&D communities, early HTA is not widely applied amongst innovators. Drawing on the experience from a workshop conducted for a group of innovators in Singapore, this work presented a case study showing how to use early HTA to set priorities, inform TPPs, and design clinical studies. We proposed deterministic targets, probabilistic-average targets, and stochastic ideal target for defining TPPs, which links the innovation process with the decision-making criteria by HTA agencies and government. Targeting audiences from R&D communities, this work aims to translate early HTA into a practical tool and promote the application of early HTA amongst R&D communities.

## CRediT authorship contribution statement

**Yi Wang:** Conceptualization, Methodology, Formal analysis, Writing – original draft, Writing – review & editing. **Waranya Rattanavipapong:** Methodology, Writing – original draft, Writing – review & editing. **Yot Teerawattananon:** Conceptualization, Methodology, Writing – original draft, Writing – review & editing, Supervision, Funding acquisition.

## Declaration of Competing Interest

The authors declare that they have no known competing financial interests or personal relationships that could have appeared to influence the work reported in this paper.

## Appendix Supplementary materials

Image, application 1

Image, application 2

Image, application 3

Image, application 4
