# Supplementary material for: Using health technology assessment to set priority, inform target product profiles, and design clinical study for health innovation
Source: Technol Forecast Soc Change. 2021 Nov;172:121000. doi: 10.1016/j.techfore.2021.121000 (PMC8524319; doi:10.1016/j.techfore.2021.121000)
Supplement: Supplementary file 1 [file mmc1.docx]

**Supplementary Material 1**

**Section A**

This section explains the supplementary R^®^ files used to generate the results in this work. Additional information on the method was provided where necessary.

**Cost-utility analysis, probability sensitivity analysis, and one-way sensitivity analysis**

Deterministic cost-utility analysis, probability sensitivity analysis, and one-way sensitivity analysis were available in *mmc2*.

**Target Product Profiles**

Calculations of cost of innovator’s technology I satisfying minimally acceptable target and stochastic ideal target achieving 90% were presented in *mmc3.* We used manual search to identify the target value. Optimization solver can also be applied to find the target value. A dataset of simulated parameter values were provided in *mmc4* for demonstration.

**Value-of-information analysis**

We employed similar steps as in Ades et al. (2004) to calculate expected value of perfect information (EVPI), expected value of partial perfect information (EVPPI), and expected value of sample information (EVSI).

For EVSI calculation, one issue is that cost of I followed Gamma distribution in the original setup. EVSI calculation will be complicated and time-consuming using the original distribution. We used Log-normal distribution to approximate Gamma distribution by matching the mean and variance. The plot of density functions of the two distributions were presented in Figure S1. They are close to each other.

Figure S1: Density Functions of Cost of I in Orignal Gamma Distribution and Approxiated Log-normal Distribution


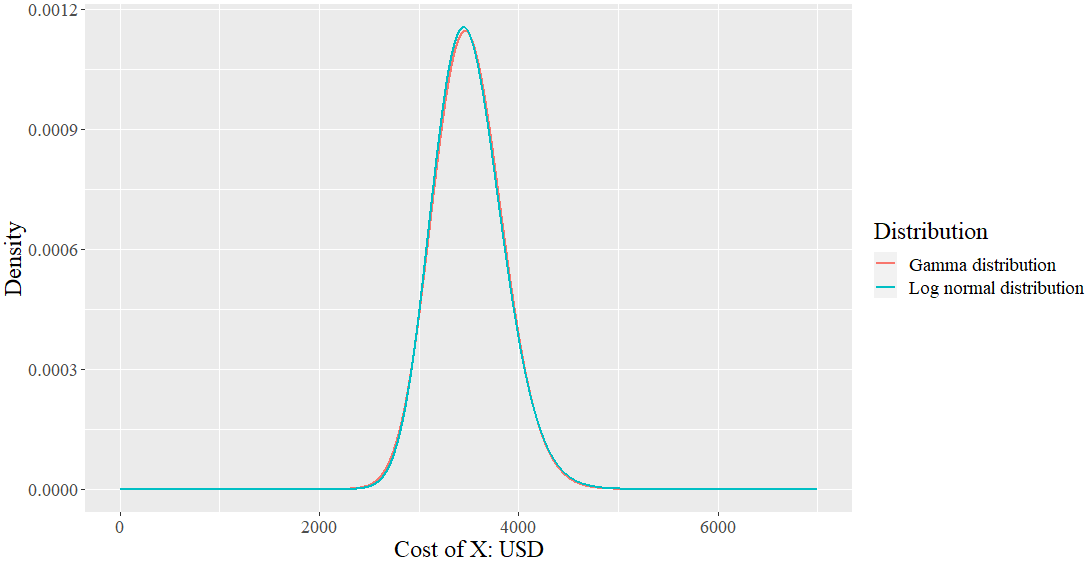


Following the method proposed by Ades et al. (2004), we also calculated the EVSI considering the scenario of known variance and unknown variance with the log-normal distribution. The results for known variance are the same as results in Table 5.

Figure S2: EVSI – Cost of Technology I


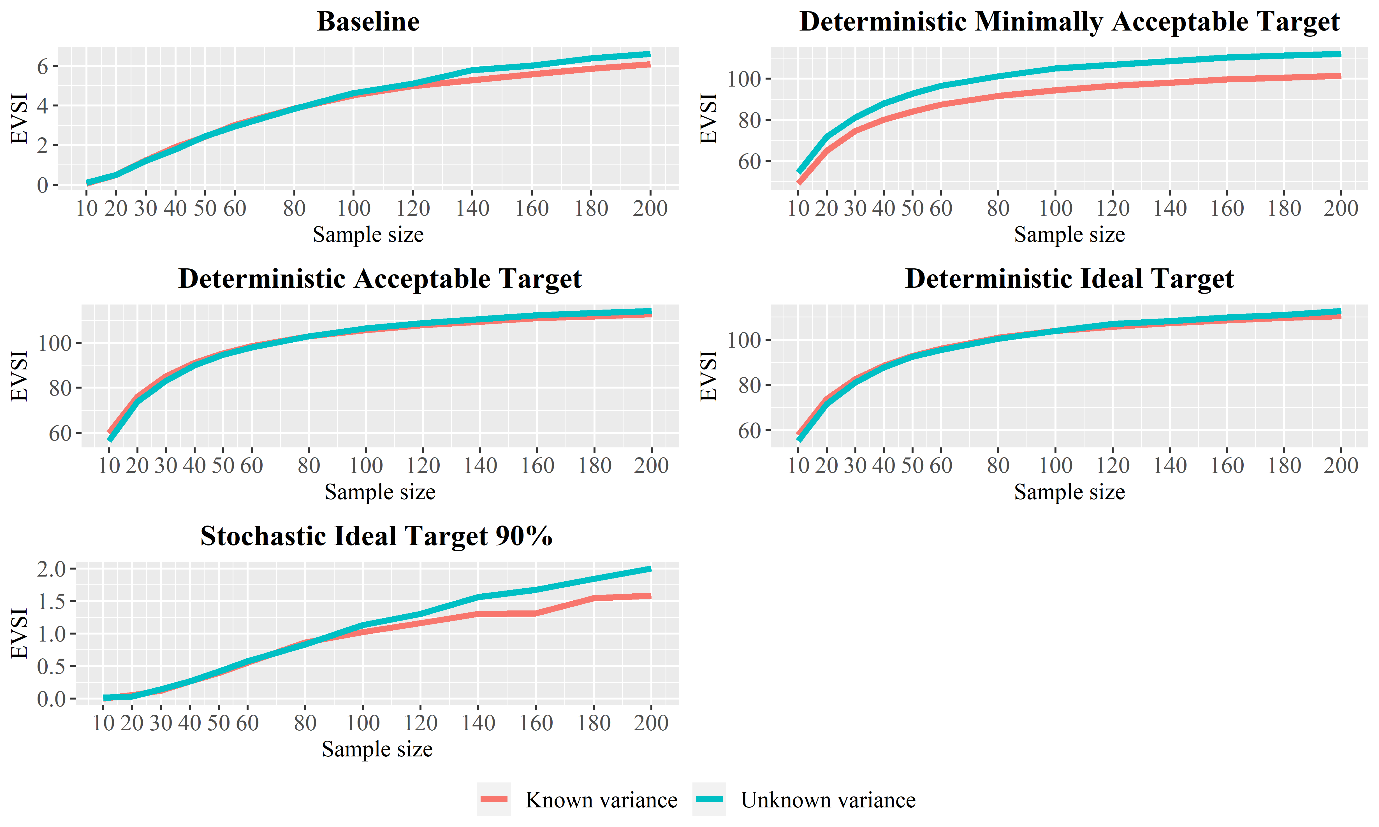


**Section B**

In our study, TPP calculation mainly considers improving the average performance focusing on the mean of the parameters. However, in reality, stability of the performance could also be relevant, especially during early stage of innovation. In such a case, it will be appropriate to model a certain feature of the technology using a distribution. Assume the distribution can be categorized by a mean parameter and a variance parameter. Considering the example below:

| Parameter | Mean | SE |
| --- | --- | --- |
| Scenario A: parameter being a fixed value | | |
| Relative risk of complication: mean | 0.8 | 0.3 |
| Scenario B: parameter following a distribution | | |
| Relative risk of complication: mean | 0.8 | 0.2 |
| Relative risk of complication: variance | 0.25*0.25 | 0.1 |

Scenario A is the setup of the current study, modelling the mean of treatment outcome. Scenario B considers the treatment outcome following a distribution. The variance parameter could measure the stability of the performance of the technology or could be due to certain unobserved heterogeneity at the population level. It could also be due to the technology being new and immature, a lack of quality control measures, or considering different treatment regimes. The variance parameter is different from standard error (SE). The variance parameter obtained from clinical or observational studies could have SE as well. SEs are not part of the product profile and are related to the sampling issues, e.g.: small sample size, in clinical studies. SE can be reduced by conducting additional research in VOI analysis. Variance can be a product profile, e.g.: stability of performance, and can be improved by additional R&D. Both variance and SE can cause decision uncertainties, but they should be addressed in different ways.

Considering additional variance parameter has no impact on calculating deterministic targets, unless the outcome measures are affected by variance parameter directly. However, to achieve probabilistic-average targets and stochastic ideal target, innovators may consider changing either mean parameter, e.g.: the average performance of the innovation, or variance parameter, e.g.: the stability of the performance of the innovation. The innovator may also consider changing these two parameters together using the same method as the two-way analysis to determine the TPPs.

In general, it is straightforward to incorporate variance parameter to calculate the proposed TPPs. The variance parameter should be incorporated in the model explicitly. Same methods of calculating TPPs can be applied. However, empirically, there could be some technical challenges. For example, certain strong assumptions, e.g.: distribution assumptions and model assumptions, are required to separately identify mean of variance (e.g.: 0.25*0.25) and SE of mean (e.g.: 0.2). Furthermore, this will also affect the VOI calculation.

**Section C**

We show a hypothetical example below demonstrating that achieving stochastic ideal target with 90% could lead to both low- and high-VOI. This is to show that VOI analysis could still be important under the stochastic ideal target with 90%.

|  | **SIT 90%: low VOI** | **SIT 90%: high VOI** |
| --- | --- | --- |
| 30% | 12 | 12 |
| 30% | 11 | 11 |
| 30% | 10 | 10 |
| 10% | -10 | -90 |
| Probability of cost effective | 90% | 90% |
| Expected net monetary benefit | 8.9 | 0.9 |
| VOI | 1 | 9 |

We considered two scenarios. Under both scenarios, we assume the net monetary benefit being 12, 11, 10 with 30% of chance each. In the first scenario, we assume there is 10% of chance of -10 loss. In the second scenario, we assume there is 10% of chance of -90 loss. Both scenarios satisfy stochastic ideal target with 90% and have positive expected net monetary benefit. However, VOI is much higher for the second case compared to the first case.

Reference:

Ades, A.E., Lu, G., Claxton, K., 2004. Expected Value of Sample Information Calculations in Medical Decision Modeling. Med. Decis. Mak. 24, 207–227. https://doi.org/10.1177/0272989X04263162
